# Pure testicular choriocarcinoma with gastrointestinal metastasis and paraneoplastic symptoms: a case report

**DOI:** 10.1186/s12894-023-01271-0

**Published:** 2023-06-03

**Authors:** Negin Namavari, Lohrasb Taheri, Farhang Hooshmand, Mohammad H. Dousthaghi, Vahid Rahmanian

**Affiliations:** 1 School of Medicine, Peymaniye Hospital, Jahrom University of Medical Science, Jahrom, Iran; 2 Department of Surgery, Jahrom University of Medical Science, Jahrom, Iran; 3 Department of pathology, School of Medicine, Jahrom University of Medical Sciences, Jahrom, Iran; 4 Biosystems Engineering Faculty, Tarbiat Modares University, Tehran, Iran; 5 Department of Public Health, Torbat Jam Faculty of Medical Sciences, Torbat Jam, Iran

**Keywords:** Acute abdomen, Gastrointestinal metastases, Testicular choriocarcinoma, Melena, Paraneoplastic symptoms, Case report

## Abstract

**Background:**

Pure testicular choriocarcinoma is a rare type of non-seminomatous germ cell tumor extremely poor prognostic with the tendency to bleed at the metastatic site. At the time of the diagnosis, 70% of patients have metastatic lesions. Depending on the site of the metastasis, symptoms vary. Gastrointestinal involvement is seen in less than 5% of cases, mostly in the duodenum.

**Case presentation:**

We present a 47 years old male with testicular choriocarcinoma involving the jejunum, lung, liver, and kidney presenting with acute abdominal pain, melena, and dyspnea with some paraneoplastic symptoms. The patient had increased, severe and constant pain in the right lower quadrant for the previous four days. Additionally, he was complaining of nausea, vomiting, anorexia, and a history of melena for the last 10 days. Dyspnea on exertion, hemoptysis, and dry cough were the symptoms he was suffering from, for almost one year. The patient’s general appearance was pale, ill, and thin with 10 kg of weight loss during the last some months. The computed tomography (CT) scan reported multiple metastatic lesions in both liver lobes and the left kidney. Pathologic study of the samples of small bowel lesions showed metastatic choriocarcinoma. Following the patient had been referred to an oncologist to start the chemotherapy regime. Finally, the patient has expired after 40 days of his first admission.

**Conclusions:**

Testicular choriocarcinoma is a rare but fatal malignancy among young men. Gastrointestinal metastases are infrequent involvement represented by melena and acute abdominal pain, obstruction, and mass. Physicians should consider it as a differential diagnosis for acute abdomen and gastrointestinal bleeding causation.

## Background

Testicular cancer is the most common cancer in young men, however, it is a rare cancer among general population [1]. The gradual increase of its incidence [2] makes it more important than ever. According to GLOBOCAN 2020 report, age-standard rate (ASR) for testicular cancer in world was 1.8 /100,000. The highest incidence rate were in Europe (west-north and south parts) and Oceania with ASR 7 followed by north America :5.6 and the lowest ASR belonged to Asia and Africa:2 [3].Testicular choriocarcinoma is the rarest non-seminomatous germ cell tumor [4]. The cancer has an aggressive behavior which has the worst prognosis of all types of testicular germ cell tumors [5], however, the lack of specific symptoms of it, makes the diagnosis difficult [6]. At the time of diagnosis, almost 70% of the patients have metastatic disease [4]. Early metastases to lung and liver is one of the typical characteristics of this tumor [7]. Such symptoms of gastrointestinal are extremely rare [8]. We report a case of testicular choriocarcinoma involving small intestine, lung, liver and kidney presenting with acute abdominal pain, melena and dyspnea with some paraneoplastic symptoms.

## Case presentation

### History and physical examination

This is a case report of a 47 years old male with chief compliant of acute abdominal pain who came to the emergency department. As past medical illness he mentioned a history of recent hyperthyroidism however he didn’t consume any drug. Prior hospitalizations, family history and allergy history were negative. The patient had 35 pack-year smoking history. He was suffering from dyspnea on exertion, hemoptysis and dry cough, for almost one year. In present illness he experienced nausea, vomiting, anorexia and melena for last ten days. He was complaining of an increasing, severe and constant pain on right lower quadrant for previous four days. The general appearance of the patient was pale, unhealthy, and skinny after losing ten kilograms over the past few months. Findings of physical examination included pale mucosa, no cervical and auxiliary lymphadenopathy, decrease in right lung sound, normal heart sound S1-S2, soft abdomen with right lower quadrant tenderness and rebound tenderness, bilateral gynecomastia, no chest pain, bilateral atrophic testis, no inguinal lymphadenopathy.

#### Diagnosis and course of Disease

Pre-operation laboratory data shown 7.5 milligram per deciliter (mg/dl) hemoglobin which transfused by two unit of packed red blood cell and pantoprazole. Laboratory tests of the patient are reported in Tables 1 and 2.

**Table 1:** Biochemistry test results

Measure Parameter (Unit)	Amount
White Blood Cell (/µLitre)	11.3
Platelets (/µLitre)	323,000
Blood Urea Nitrogen (milligram/Decilitre)	9
Blood Sugar (milligram/Decilitre)	74
Fasting Blood Sugar (milligram/Decilitre)	99
Creatinine (milligram/Decilitre)	0.8
Sodium (milliequivalent/Litre)	132
Potassium (Millimole/Litre)	3.6
Partial Thromboplastin time (Second)	43
Prothrombin Time Test (Second)	21
International Normalized Ratio	1.57
Lactic Acid Dehydrogenise (Unite/Litre)	2126
Albumin (Gram/Decilitre)	2.3
C-reactive Protein (milligram/Litre)	115
Erythrocyte Sedimentation Rate (millimetre/Hour)	32
**Liver function test**	
Tested Parameter	Amount
Aspartate Aminotransferase (Unit/Lt)	116
Alanine Aminotransferase (Unit/Lt)	14
Alkaline Phosphatase (International Unite/Lt)	450
Total Bilirubin (milligram/Decilitre)	2.2
Direct Bilirubin (milligram/Decilitre)	1.8

**Table 2:** plural fluid analysis

Measure Parameter (Unit)	Amount
Lactic Acid Dehydrogenise (Unite/Litre)	19,809
Albumin (Gram/Decilitre)	5.2
Blood Sugar (milligram/Decilitre)	66

**Fig. 1:**
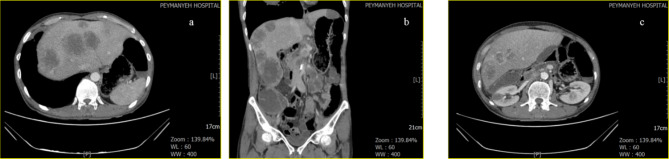
Abdominopelvic CT scan a, b and c) Axial, coronal and axial view depicting multiple lesions in liver and kidney

**Fig. 2:**
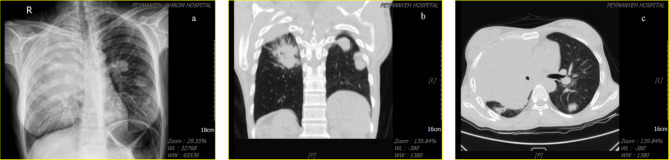
(a) Posteroanterior Chest X-ray, (b) coronal view of spiral chest CT, (c) axial view of spiral chest CT depicting multiple hemorrhagic cannonball lesions in lungs

The patient had been asked for abdominal sonography and abdominopelvic computed tomography (CT) scan which reported a multiple metastatic lesion in both liver lobes and left kidney (Fig. 1). Moreover, chest X-ray and spiral CT scan, had been asked (Fig. 2) which reported multiple bilateral lung metastatic lesion with pressure effect on right side bronchus and mediastinum.

**Fig. 3:**
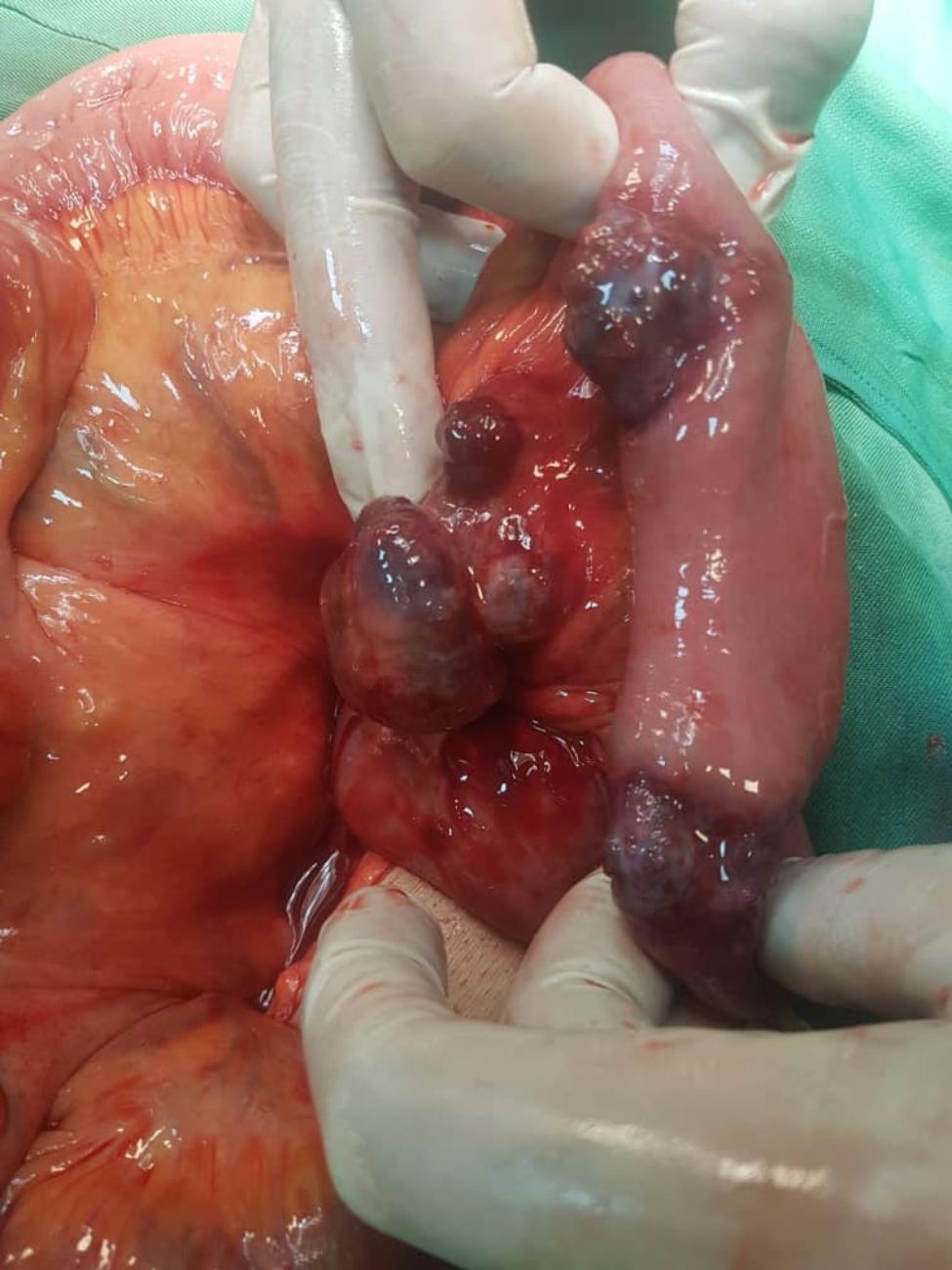
Resected a part of small bowel (jejunum) with multiple masses

Emergency laparotomy was done which showed small bowel obstruction with multiple mass-like lesion (Fig. 3) inside the lumen in 40 centimeters distant of Treitz ligament. Resection and end to end anastomosis was done.

The samples of the small bowel lesion were sent for pathologic study. He came with symptoms of constipation, vomiting, melena and abrupt hemoglobin fall to 5.6 mg/dl after two weeks of discharge. He had been cared conservatively and receive four pack cells.

**Fig. 4:**
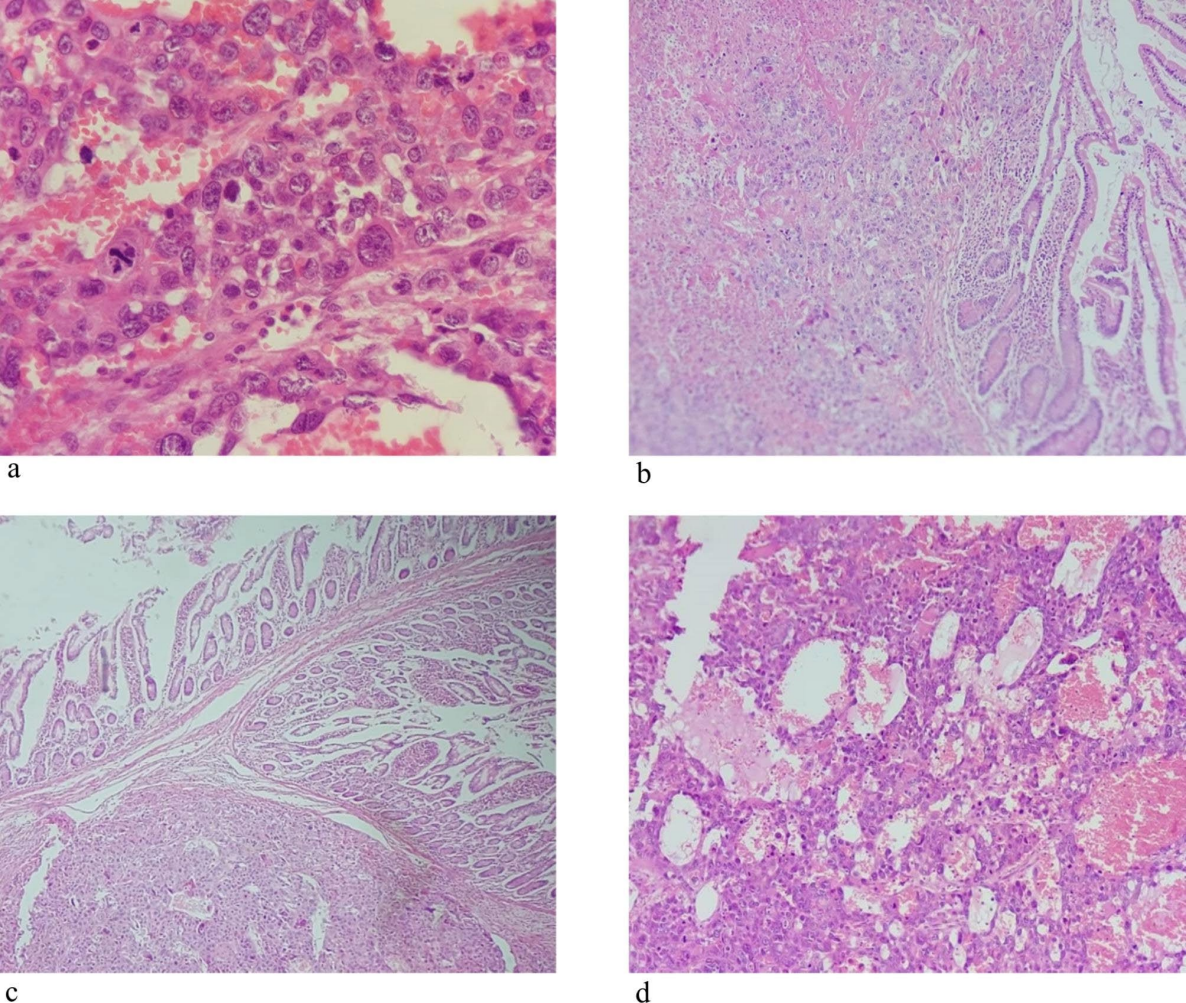
Microscopic slides demonstrate solid sheets of atypical cytotrophoblast and syncytiotrophoblast with infiltrative and destructive pattern and high mitotic activity in a background of necrosis and hemorrhage. (a) Tumoral cells exhibiting pleomorphism, extensive hemorrhage and blood-filled cystic spaces (b) Extensive involvement of mucosa with tumoral cells with lakes of hemorrhage separating tumoral cells (c) Intestinal mucosa is infiltrated by anaplastic tumoral cells replacing the bowel muscular wall extending up to muscularis mucosa. Both cytotrophoblast and multinucleated syncytiotrophoblast are depicted in the field. (d) Sheets of highly anaplastic cells with a mitosis at the center of the field

Pathologic study of the samples of small bowel lesions showed metastatic choriocarcinoma (Fig. 4). The patients β- human chorionic gonadotropin (βHCG) level was elevated at 150,000 IU/ml. serum alpha fetoprotein (AFP) level was normal at 1.45 IU/ml. Following the patient had been referred to oncologist. According to our follow-up, the patient was not able to start chemotherapy regime due to his suffering gastrointestinal symptoms. Unfortunately, he has expired after 40 days of his first admission.

## Discussion and conclusion

The case of present study is a metastatic pure testicular choriocarcinoma presented with acute abdominal pain, gastrointestinal bleeding, some paraneoplastic syndromes secondary to high titer βHCG such as bilateral gynecomastia and hyperthyroid state.

As a none-seminoma germ cell tumor (NSGCT), choriocarcinoma places among rapid progressive cancer which has not specific clinical symptoms in the beginning, while at the time of diagnosis 70% of the patients have metastatic diseases [9]. Not more than 8% of GCT cases contains component of choriocarcinoma, even more, less than 1% of them are among pure choriocarcinoma [10]. Histologically, it contains two type of cells including cytotrophoblast and syncytiotrophoblast [11] detected in the case of present study. As the most aggressive type [7]. Emanated from its excessive vascularity, it tends to hemorrhage at the site of metastases [7].

As only 5% of choriocarcinoma case has gastrointestinal tract metastasis [12] Duodenum is the most common metastatic site due to its anatomic adjacency to the retroperitoneal nodes. jejunum, ileum, stomach, esophagus, colon and pancreas are other sites which may have been involved [13]. GIT involvement is extremely rare representing with melena, hematemesis, anemia, intussusception, perforation [14], and pseudo-obstruction due to polypoidal growth [15]. Considering the gastrointestinal manifestations, and some other symptom such as abdominal pain and obstruction demonstrations of metastatic testicular choriocarcinoma, it may have been diagnosed as appendicitis, mistakenly. Therefore, as a differential diagnosis, it has to kept in mind, patients with above-mentioned symptoms, particularly in young men, testicular tumor has to be ruled out by genitourinary physical examination, testicular ultrasound sonography, serum biomarkers measurement (βHCG, (AFP), lactate dehydrogenase (LDH)), imaging of chest, abdomen and pelvis, brain magnetic resonances imaging (MRI) evaluating intracranial hemorrhage. Treatment approach depends on disease stage. If there were not metastatic evidence, it might be in early-stage indicating radical orchiectomy and lymph node dissection. Otherwise, any metastatic involvement needs chemotherapy-based cisplatin. To monitor response to treatment serial serum βHCG level check is suggested [16].

In one hand, One of most common clinical features of pure choriocarcinoma which associated with hemorrhage at metastatic sites is high level of serum (βHCG) [17] known as choriocarcinoma syndrome characterized by haemopstysis, dyspnea, hematemesis, melena and anemia [9]. On the other hand, high level of βHCG causes paraneoplastic syndromes such as hypothyroidism [18], and Gynecomastia [19]. High titer of HCG causes hyperthyroidism due to cross-reaction with thyrotropin stimulating hormone (TSH) [18] which could be related to our patient hyperthyroidism state. Moreover, the rising HCG, as LH analogue, plays as the main stimuli for Leydig cells to secrete more estrogen and less testosterone justifying the gynecomastia [19] and atrophic testis in present case. Almost 1% of testicular cancer patients present by Gynecomastia [20].

According to European Association of Urology guideline 2022, the current case has most of poor-prognosis group criteria including βHCG > 50,000 IU/ml, LDH > 10×ULN (Upper Limit Normal), the non-pulmonary visceral metastases, and mediastinal primary involvement. Consequently, the suggested treatment is either four cycles of BEP (Bleomycin, Etoposide, Cisplatin) or 4 cycles of PEI (Cisplatin, Etoposide, Ifosfamide) with 21 days intervals [21].

Our case report highlights the rare occurrence of pure testicular choriocarcinoma with gastrointestinal metastasis and paraneoplastic symptoms. Given the aggressive nature of this malignancy and the potential for distant metastasis, prompt diagnosis and treatment are critical for achieving favorable outcomes. Furthermore, the complex clinical presentation of paraneoplastic symptoms should not be overlooked, as they may be the first sign of an underlying malignancy. Clinicians should consider a thorough workup for paraneoplastic syndromes in patients with unexplained symptoms, particularly those with a history of cancer. Finally, this case underscores the need for ongoing research into the pathogenesis and optimal management of testicular choriocarcinoma, particularly in cases with atypical presentations and metastases.

One of the limitations of this study is that we didn’t have any chance to treat the patient. To the best of our knowledge, a case with all mentioned symptoms has not ever been reported, thus, its report may improve diagnosis criteria.

Testicular choriocarcinoma is a rare but fatal malignancy among young men. Gastrointestinal metastases are infrequent involvement represented by melena and acute abdominal pain, obstruction and mass. Physicians should consider it as a differential diagnosis for acute abdomen and gastrointestinal bleeding causation. Evaluating any paraneoplastic symptoms like change in hormonal state, hyperthyroidism, gynecomastia, atrophic testis by review of systems and genital examination may lead to early diagnosis and better management. However, these patients generally have poor outcome due to aggressive behavior of tumor.

## Data Availability

All data generated or analyzed during this study are included in this published article.

## Abbreviations

CT: Computed tomography
AFP: Alpha-fetoprotein
βHCG: β- Human chorionic gonadotropin
NSGCT: None-seminoma germ cell tumor
TSH: Thyroid-stimulating hormone
LDH: Lactate dehydrogenase
MRI: Magnetic Resonance Imaging
